# Spatial control of PEG-lipid interfaces at cell surface enables on-demand adhesion and enzymatic detachment of suspension cells

**DOI:** 10.1080/14686996.2026.2713991

**Published:** 2026-08-07

**Authors:** Zhihan Yang, Yuya Sato, Yuji Teramura, Tadashi Nakaji-Hirabayashi

**Affiliations:** a Master’s/Doctoral Program in Life Science Innovation (T-LSI), University of Tsukuba, Tsukuba, Ibaraki, Japan; b Cellular and Molecular Biotechnology Research Institute (CMB), National Institute of Advanced Industrial Science and Technology (AIST), Tsukuba, Ibaraki, Japan; c Faculty of Engineering, Academic Assembly, University of Toyama, Toyama, Japan; d Graduate School of Science and Engineering, University of Toyama, Toyama, Japan; e Interdisciplinary Graduate School of Medicine, Pharmacy, Science and Engineering, University of Toyama, Toyama, Japan

**Keywords:** Cell adhesion, detachment, PEG-lipid, cell surface modification, cell-penetrating peptide, Tat peptide

## Abstract

Suspension cells are widely used in biomedical research and cell-based therapies; however, their lack of stable adhesion to substrates limits their efficient handling, including gene delivery and downstream processing. Although surface engineering approaches have been studied to induce cell adhesion, precise control over attachment and detachment, comparable to that of adherent cells, remains challenging. Here, we report a strategy to control cell adhesion and detachment using enzyme-responsive trans-activator of transcription peptide–poly(ethylene glycol)-lipid (Tat-PEG-lipid) conjugates and non-functional poly(ethylene glycol)-lipids (PEG-lipid). A collagenase-cleavable peptide sequence was inserted between the Tat peptide and the PEG chain, yielding Tat-Col-PEG-lipid constructs with PEG molecular weights of 20 and 40 kDa. These conjugates were incorporated into the cell membranes of CCRF-CEM cells, a human T-lymphoblastoid cell line, to induce adhesion via the Tat peptide. To regulate the interfacial structure, Tat-Col-PEG-lipid was co-assembled with a non-functional PEG(5k)-lipid, enabling precise control over the surface density to improve collagenase access. We demonstrated that the mixed PEG-lipid modification allows robust cell adhesion to substrates while enabling efficient detachment upon collagenase treatment. Notably, the use of Tat-Col-PEG-lipids with longer PEG chains (40 kDa) significantly improved the adhesion efficiency, enzymatic detachment, and cell viability compared to shorter PEG chains. Optimal mixing ratios of Tat-Col-PEG(40k)-lipids and PEG(5k)-lipids resulted in stable attachment and rapid collagenase-triggered release across different substrates, planar surfaces, and fibrous scaffolds. This hierarchical PEG-lipid modification approach provides a versatile and minimally invasive platform for the transient manipulation of suspended cells, with potential applications in gene delivery, cell processing, and regenerative medicine.

## IMPACT STATEMENT

This study establishes an enzyme-responsive PEG-lipid interface that enables transient adhesion and viable recovery of suspension cells for cell processing and regenerative medicine applications.

## GRAPHICAL ABSTRACT

**Figure:**
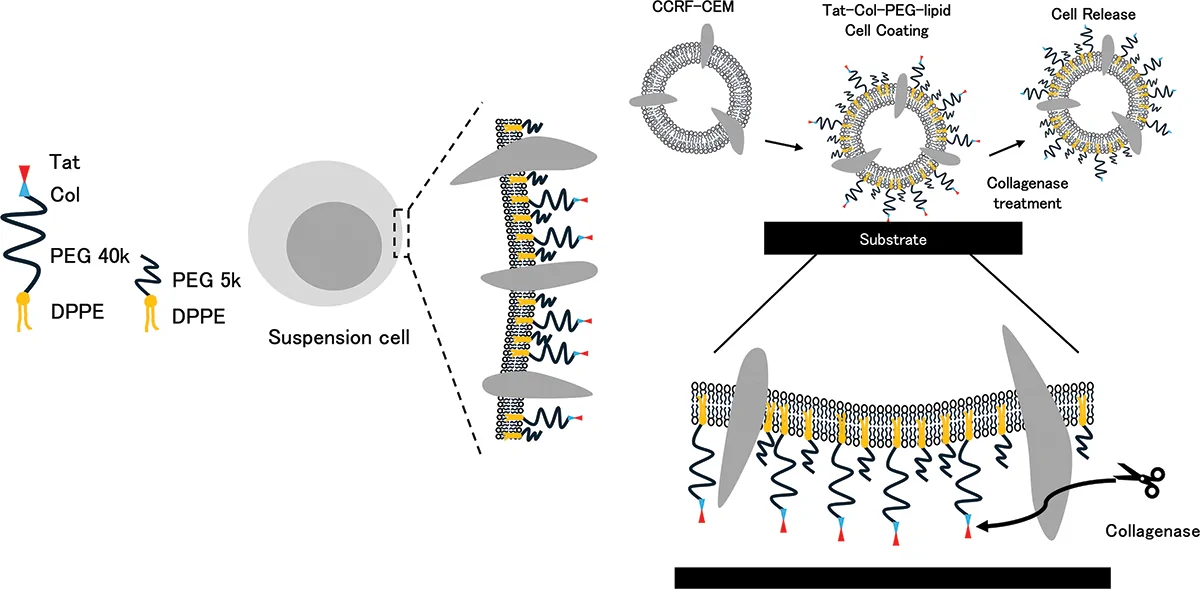

## Introduction

Suspension cell lines of hematopoietic origin are important model systems for immunology and hematologic cancer research because they provide self-renewing, accessible, and experimentally tractable human cells for studying immune-cell biology and pharmacological responses; among these, CCRF-CEM is a well-established suspension human T-lymphoblast cell line derived from acute leukemia [1,2]. Similar lymphoid and myeloid suspension cells are widely used in immunological studies as well as in gene delivery and drug response investigations [1,3–5]. In particular, non-adherent immune cells are important targets for nucleic acid delivery and genome engineering; however, they remain among the most difficult cell types to transfect with non-viral systems, exhibiting low efficiency and substantial toxicity [3,6]. Unlike adherent cells, suspension cells lack stable substrate adhesion, which limits vector–cell interactions and thereby reduces transfection efficiency [3,4,6,7]. Therefore, transient immobilization or capture of suspension cells on substrate surfaces is important for improving transfection. Several strategies have been explored to address these limitations, including genetic engineering of surface receptors, immobilization via antibody or ligand coatings, and chemical tethering using polymers or nanomaterials [8–10]. However, these approaches may involve complex procedures, limited flexibility, or potential effects on cell phenotype and viability, posing challenges for clinical translation [8–13]. In addition to transient immobilization, mild and efficient cell harvesting from substrates is required after transfection, but conventional harvesting often requires additional treatment steps that increase process complexity. These constraints motivated us to develop a strategy for modulating the adhesion and detachment of suspension cells while preserving cellular function.

Poly(ethylene glycol)-lipid (PEG-lipid) conjugates offer a promising approach for non-genetic surface engineering [14,15]. The lipid domain is inserted into the lipid bilayer membrane, whereas the PEG chain extends into the aqueous phase and can carry functional groups, enabling the functionalization of the cell surface under mild conditions [16,17]. Cell surface modification with PEG-lipid conjugates has been used to anchor polymers, proteins, oligopeptides, and oligonucleotides to live cells and to regulate specific cell–cell or cell–surface interactions [14–20]. We found that conjugation with cell-penetrating peptides (CPPs) such as the HIV-1 Tat basic domain further broadened the applicability of cell modification [21,22]. Tat and related arginine-rich peptides readily translocate across negatively charged cell membrane [23,24]. In our previous study, however, when we used Tat-PEG-lipids for cell surface modification, the modified cells spontaneously adhered to the substrate surface because of the electrostatic interactions between the positively charged CPPs and the negatively charged substrate [21,22]. Therefore, Tat-PEG-lipid conjugates provide a facile and non-invasive method to induce the adhesion of suspension cells. Recently, we introduced collagenase-responsive linkers (collagen-derived peptide sequences, Col) into Tat-PEG-lipids, in which the Tat peptide was linked to PEG chains via Col, and examined the collagenase responsiveness of Tat-Col-PEG-lipids using liposomes as a model cell system [25]. Liposomes modified with Tat-Col-PEG-lipids spontaneously adhered to the substrate surface and the adhered liposomes were detached by collagenase treatment. These results provide a proof of concept for the enzyme-responsive control of adhesion and detachment.

**Scheme 1.**
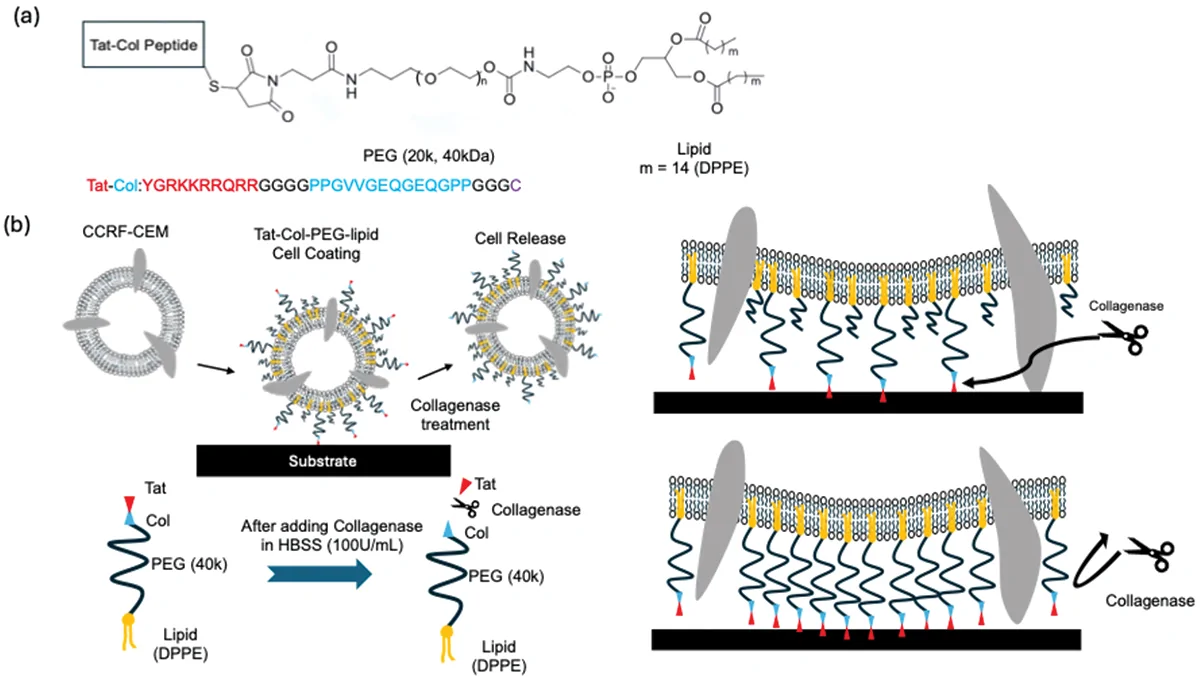
Schematic illustration of transient CCRF-CEM cell adhesion and collagenase-mediated release using Tat-Col-PEG-lipid surface modification. (a) Tat-Col-PEG-lipid consists of a Tat peptide, a collagenase-cleavable Col linker, a PEG spacer chain, and a DPPE lipid anchor. (b) Transient cell adhesion and collagenase-mediated release. The lipid moiety inserts into the CCRF-CEM cell membrane, while the PEG chain presents the Tat peptide away from the cell surface. The exposed Tat peptide promotes adhesion of normally suspension CCRF-CEM cells to the substrate. After treatment with collagenase in HBSS, the Col linker is cleaved, reducing Tat-mediated cell–substrate interaction and allowing cell detachment. Co-insertion of non-functional PEG(5k)-lipid adjusts the spacing of Tat-Col-PEG-lipid on the membrane surface and improves collagenase accessibility to the cleavable linker.

In this study, we aimed to optimize cell adhesion and subsequent detachment through the surface modification of suspension cells, CCRF-CEM using Tat-Col-PEG-lipid conjugates and collagenase. We compared the Tat-Col-PEG-lipid conjugates bearing PEG chains of different molecular weights (20 and 40 kDa) with C16 lipid anchors (Scheme 1). These constructs were used to modify the surfaces of CCRF-CEM cells. In addition, a non-functional PEG-lipid with a shorter PEG chain of 5 kDa was co-inserted to optimize collagenase access.

We hypothesized that non-functional PEG-lipids would serve as spacers between Tat-Col-PEG-lipids because a large enzyme such as collagenase (ca. 100 kDa) may have limited access to the Col domain when the cell surface is densely occupied with Tat-Col-PEG-lipids. To improve access to the Col domain, we reasoned that a shorter PEG chain (5 kDa) would create effective spacing between adjacent Tat-Col-PEG-lipids. To test this hypothesis, the cells were modified with varying ratios of Tat-Col-PEG-lipids and PEG (5k)-lipids, and their adhesion to substrates and collagenase-triggered detachment were systematically evaluated. Furthermore, we examined the effects of PEG chain length and mixed PEG-lipid composition on adhesion efficiency, enzymatic detachment, and cell viability and extended the analysis to both planar and fibrous substrates (Scheme 1).

## Materials and methods

### Materials

Poly(ethylene glycol) (PEG) derivatives with maleimide and N-hydroxysuccinimide ester functionalities (Mal-PEG-NHS, MW 5, 20 and 40 kDa), 1,2-dipalmitoyl-*sn*-glycero-3-phosphoethanolamine (DPPE) and poly(2-methacryloyloxyethyl phosphorylcholine (MPC)-co-n-butyl methacrylate) with a 0.30 MPC unit mole fraction (MPC polymer) were obtained from NOF Corporation (Tokyo, Japan). Tat (YGRKKRRQRRRC), Tat-Col (YGRKKRRQRRRGGGGPPGVVGEQGEQGPPC), and FITC-Tat-Col were purchased from Sigma-Aldrich (St. Louis, MO, U.S.A.) and used without further purification. Rhodamine-GC was purchased from BEX Co. Ltd. (Tokyo, Japan). Glass-bottomed dishes were purchased from Matsunami Glass Industries (Osaka, Japan). Collagenase type IV (290 U/mg) was obtained from Worthington Biochemical Corporation (Lakewood, NJ, U.S.A.). RPMI-1640 medium, Hank’s balanced salt solution (HBSS), antibiotics (penicillin and streptomycin), diethyl ether, triethylamine, chloroform, methanol, acetone, super-dehydrated dichloromethane, sodium chloride (NaCl), dimethyl sulfoxide (DMSO), ethanol, sucrose, cholesterol, and Dulbecco’s phosphate-buffered saline (PBS; pH 7.4) were purchased from FUJIFILM Wako Pure Chemical Corporation (Osaka, Japan). Fetal bovine serum (FBS) was supplied by Gibco (Thermo Fisher Scientific, Waltham, MA, U.S.A.). CCRF-CEM cells, a human acute lymphoblastic leukemia T-cell line, were obtained from the Health Science Research Resources Bank (Tokyo, Japan). 24-well tissue culture polystyrene (TCPS) plates were obtained from Iwaki (Asahi Glass Co., Ltd., Tokyo, Japan). Pierce™ polyacrylamide spin desalting columns (7 K MWCO), CellTrace™ Oregon Green™ 488 carboxy-DFFDA, and trypan blue solution was purchased from Invitrogen (Thermo Fisher Scientific Waltham, MA, U.S.A.). Triton X-100 was obtained from Sigma-Aldrich (St. Louis, MO, U.S.A.).

## Methods

### Cell culture

CCRF-CEM cells were used in this study because of their suspension growth characteristics. Cells were maintained in RPMI-1640 medium supplemented with 10% FBS, 100 U/mL penicillin, and 100 µg/mL streptomycin. Culture was performed in a humidified incubator at 37°C with 5% CO_2_ and 95% air.

### Coating plastic and glass materials with MPC polymer

All containers, pipette tips, cuvettes, and tubes were coated with the MPC polymer to suppress the nonspecific adsorption of Tat-Col-PEG-lipids onto their surfaces [25]. Specifically, each container, pipette tip, cuvette, and tube were filled with a 0.5% MPC polymer solution in ethanol and rotated overnight, after which the residual solution was removed, and the surfaces were subsequently dried at room temperature.

### Synthesis of maleimide-PEG-lipids

Maleimide-PEG-lipids (Mal-PEG-lipids) with various PEG molecular weights (5, 20, and 40 kDa) and DPPE were synthesized as previously described [16,17] Briefly, Mal-PEG-NHS (5, 20, or 40 kDa; 100 mg each) was reacted with DPPE (15, 4, and 2 mg, respectively) in dichloromethane (1.5 mL) with triethylamine (50 µL). The reaction mixture was stirred at room temperature for 24 h, followed by precipitation with diethyl ether. The resulting Mal-PEG-lipid conjugates were collected and dried under a vacuum.

### Conjugation of Tat peptide to maleimide-PEG-lipids

For subsequent functionalization, synthetic oligopeptides containing a C-terminal cysteine residue were conjugated to the maleimide moiety via thiol–maleimide coupling. Mal-PEG-lipids (20 kDa, 48 mg; 40 kDa, 108 mg) were mixed with Tat-Col (8.10 mg for the 20 kDa conjugate; 9.34 mg for the 40 kDa conjugate) or FITC-Tat-Col (9.00 mg for the 20 kDa conjugate; 10.37 mg for the 40 kDa conjugate). PBS (3.99 mL for the 20 kDa conjugate; 9.87 mL or 9.76 mL for the 40 kDa conjugate) was then added to adjust the total reaction volume to 4.8 mL and 10.8 mL, respectively. The mixtures were incubated under gentle stirring at 37°C overnight. After the reaction, the products (Tat-Col-PEG(20k, 40k)-lipid and FITC-Tat-Col-PEG(20k, 40k)-lipid) were purified by spin-column chromatography (pre-equilibrated with PBS) and collected for subsequent use.

### Rhodamine labeling of PEG-lipid

To prepare fluorescence labeled PEG(5k)-lipid solutions, the following reagents were mixed and stirred at room temperature for 24 h: Mal-PEG(5k)-lipids (1 mg, 1 equivalent), Rhodamine-B GC solution (14 µL of 10 mg/mL in DMSO, 1.1 equivalent each), and PBS (86 µL). The mixture was purified using spin column chromatography (pre-equilibrated with PBS) equilibrated with PBS to remove unbound Rhodamine B GC, thereby yielding the Rhodamine-labeled non-functional PEG(5k)-lipid (Rhodamine-PEG(5k)-lipid).

### Formulation of mixed PEG-lipids

To modulate the surface density of the functional groups on the cell membranes, Tat-Col-PEG-lipid or FITC-Tat-Col-PEG-lipid (20 kDa and 40 kDa) was mixed with PEG(5k)-lipid or rhodamine-labeled PEG(5k)-lipid at defined molar ratios. Each Tat-Col-PEG-lipid or FITC-Tat-Col-PEG-lipid solution was adjusted to a final concentration of 1 mg/mL in PBS. Subsequently, PEG(5k)-lipid was added to achieve Tat-Col-PEG-lipid: PEG-lipid molar ratios of 1:0, 1:1, 1:4, 1:8, 1:16 or 0:1, calculated based on the molecular weights of the respective PEG-lipid species. The mixtures were gently stirred to ensure complete homogenization. These mixed assemblies were subsequently employed for cell surface modification experiments to investigate the effects of PEG chain density and spacing on adhesion and enzymatic detachment.

### Cell surface modification with Tat-Col-PEG-lipids and PEG(5k)-lipids

CCRF-CEM cells (5 × 10^5^ cells) were collected by centrifugation (180 × g, 3 min, room temperature) and washed once with PBS prior to use for further experiments. After removal of the supernatant, 50 µL of the mixture of Tat-Col-PEG-lipid and PEG(5k)-lipid prepared as described above was added to the cell pellet and incubated at room temperature for 30 min with gentle agitation to allow insertion into the cell membrane. After incubation, the cells were washed once with PBS by centrifugation (180 × g, 3 min, room temperature) to remove the unbound conjugates, resuspended in complete culture medium, and cultured in a TCPS dish for subsequent evaluation. CCRF-CEM cells were treated with FITC-labeled Tat-Col-PEG-lipids and Rhodamine-labeled PEG(5k)-lipids, using the same procedure.

### Quantitative analysis of adhesion efficiency

Modified CCRF-CEM cells were seeded onto the TCPS (8 × 10^4^ cells/cm^2^) and incubated for 1 h to allow for adhesion. Non-adherent cells were removed by gentle washing twice with PBS, after which the number of firmly attached cells was determined by phase-contrast microscopy (IX83, Olympus, Tokyo, Japan). The adhesion efficiency was calculated as follows:

$$\mathrm{Adhesion}\;\mathrm{efficiency}\;\left(\%\right)=N_{\mathrm{attached}}/N_{\mathrm{seeded}}\times 100$$

where *N*_attached_ and *N*_seeded_ denote the numbers of attached and seeded cells, respectively. Quantification of the attached cells was performed using the ImageJ software (NIH, Bethesda, MD, U.S.A.) from at least five randomly selected microscopic fields per well. All adhesion assays were conducted in triplicates.

### Quantitative analysis of cell morphology

Phase-contrast images obtained during the adhesion assay were used to evaluate changes in cell morphology. The modified CCRF-CEM cells that adhered to the TCPS surfaces were observed using a phase-contrast microscope (IX83, Olympus, Tokyo, Japan) under the same conditions described above. The cell spreading area was quantified by measuring the area of individual adhered cells using the ImageJ software (NIH, Bethesda, U.S.A.). At least five randomly selected microscopic fields per well were analyzed for each condition, and the mean spreading areas were compared among the groups to assess the morphological changes associated with surface modification. All analyses were conducted in triplicates.

### Collagenase-responsive detachment of adhered cells

The adhered cells were treated with collagenase type IV following the initial adhesion assay. Briefly, 500 µL of collagenase solution (100 U/mL in HBSS) was added to each well, and the cells were incubated at 37°C for 1 h. The detached cells were collected by washing the wells five times with PBS to ensure the removal of weakly bound cells. Residual adherent cells were subsequently imaged using phase-contrast microscopy to assess cell detachment.

The detached cells were pelleted by centrifugation (180 × g, 3 min), resuspended in fresh culture medium, and transferred to untreated culture dishes overnight. Detachment efficiency was calculated as follows:

$$\mathrm{Detachment}\;\mathrm{efficiency}\;\left(\%\right)\;=\;N_{\mathrm{detached}}/N_{\mathrm{attached}}\;\times \;100$$

where *N*_detached_ is the number of cells released after collagenase treatment and *N*_attached_ is the initial number of adhered cells. All detachment assays were performed in triplicate, and cell counts were averaged across independent experiments.

### Viability of detached cells

The detached cells were cultured overnight, and live cells were quantified using the trypan blue exclusion assay. The cell counts were normalized to the initial seeding density on day 0 to account for variations in the number of starting cells. Results were reported as mean ± SD across three independent trials.

### Analysis of surface modification status through confocal microscopy observation

To visualize the surface modification, CCRF-CEM cells were modified with FITC-labeled Tat-Col-PEG-lipids together with Rhodamine-labeled non-functional PEG-lipids under the same modification conditions described above. After incubation, cells were transferred to glass-bottomed dishes and observed under a confocal laser scanning microscope (LSM700; Carl Zeiss Microscopy Co., Ltd., Jena, Germany). Fluorescence patterns were analyzed to assess whether the PEG-lipid conjugates were uniformly distributed along the cell membrane. Co-localization analysis of FITC and rhodamine signals was performed using ZEN software (Carl Zeiss) to evaluate the spatial relationship between Tat-Col-PEG-lipids and non-functional PEG-lipids.

### Quantitative fluorescence analysis

For quantitative analysis of surface modification, 100 µL of a cell suspension (2 × 10^5^ cells/mL) modified with FITC-labeled Tat-Col-PEG-lipid and Rhodamine-labeled PEG(5k)-lipid was lysed with 1 µL of Triton X-100 and incubated for 30 min with agitation. The lysates were diluted in PBS and analyzed using a fluorophotometer (DS-11 FX+; DeNovix, Wilmington, DE, U.S.A.). Standard curves constructed from known concentrations of FITC- and Rhodamine-labeled conjugates were used to estimate the average number of molecules incorporated per cell. FITC and Rhodamine fluorescence intensities in modified cells were normalized to those obtained under the 1:0 and 0:1 conditions, respectively, after correction for background signals from non-modified cells.

For quantitative analysis of enzymatic reactivity of the Col domain, cells modified under the same conditions were incubated with collagenase type IV (100 U/mL in HBSS) at 37°C for 1 h, followed by three washes with PBS to remove residual enzyme. The fluorescence from FITC-Tat-Col-PEG-lipid and Rhodamine-PEG-lipid in the post-treatment lysates were quantified using the fluorometric protocol described above. Comparison of the fluorescence signals before and after collagenase treatment enabled the assessment of cleavage efficiency.

### Zeta potential measurement

The effects of PEG-lipid incorporation and collagenase-mediated cleavage on cell surface charge were assessed using a Zetasizer Nano ZS (Malvern Instruments, Worcestershire, UK). For each condition, 2 × 10^6^ CCRF-CEM cells were modified as described above, washed with PBS, and resuspended in 0.15 M sucrose solution containing 1 mM NaCl. All the measurements were conducted under suspension conditions to avoid substrate-induced effects. The samples were loaded into DTS 1060 cuvettes, and the zeta potential was measured at room temperature. Following baseline measurements, cells were incubated in suspension with collagenase type IV (100 U/mL in HBSS, 37°C, 1 h), washed, and reassessed for zeta potential.

To evaluate the stability of surface modifications after collagenase-mediated detachment, CCRF-CEM cells were treated with Tat-Col-PEG(40k)-lipid and PEG(5k)-lipid mixtures at the indicated ratios. The cells from all ratio groups were treated with collagenase for 1 h and then cultured in medium for 12 h. The zeta potential was measured. Untreated CCRF-CEM cells were included as controls and processed in parallel under the same conditions, including collagenase treatment for 1 h, followed by cell culture. Comparisons were made among untreated CCRF-CEM cells and those treated with different ratios of Tat-Col-PEG-lipids and PEG-lipids before and after collagenase treatment.

### Evaluation of adhesion and detachment of cells on a fabric scaffold

Modified CCRF-CEM cells (8 × 10^4^ cells/well) were seeded onto fabric scaffold (Ø35 mm, ecellba®, Teijin Frontier, Tokyo, Japan) per well in a 24-well plate and incubated for 1 h to allow adhesion. Non-adherent cells were removed by gentle washing twice with PBS　and were labeled by incubating with CellTrace™ Oregon Green solution (1 µL) at 37°C for 30 min, after which, the unbound dye was washed with PBS. After washing, the cells on the fabric scaffolds were observed under a confocal laser scanning microscope.

### Statistical analysis

All experiments were conducted in triplicate, unless otherwise noted. Data are presented as mean ± SD. Statistical significance was assessed using one-way analysis of variance (ANOVA), followed by Dunnett’s post hoc test for multiple comparisons. All statistical analyses were performed using Microsoft Excel for Mac (version 16.96.1; Microsoft Corporation, Redmond, WA). Statistical significance was set at *p* < 0.05. Significance thresholds are denoted as follows: *p < 0.05, **p < 0.01, ***p < 0.001, and ****p < 0.0001.

## Results

### Co-insertion of Tat-Col-PEG(40k)-lipid and PEG(5k)-lipid into the cellular membrane

**Figure 1.**
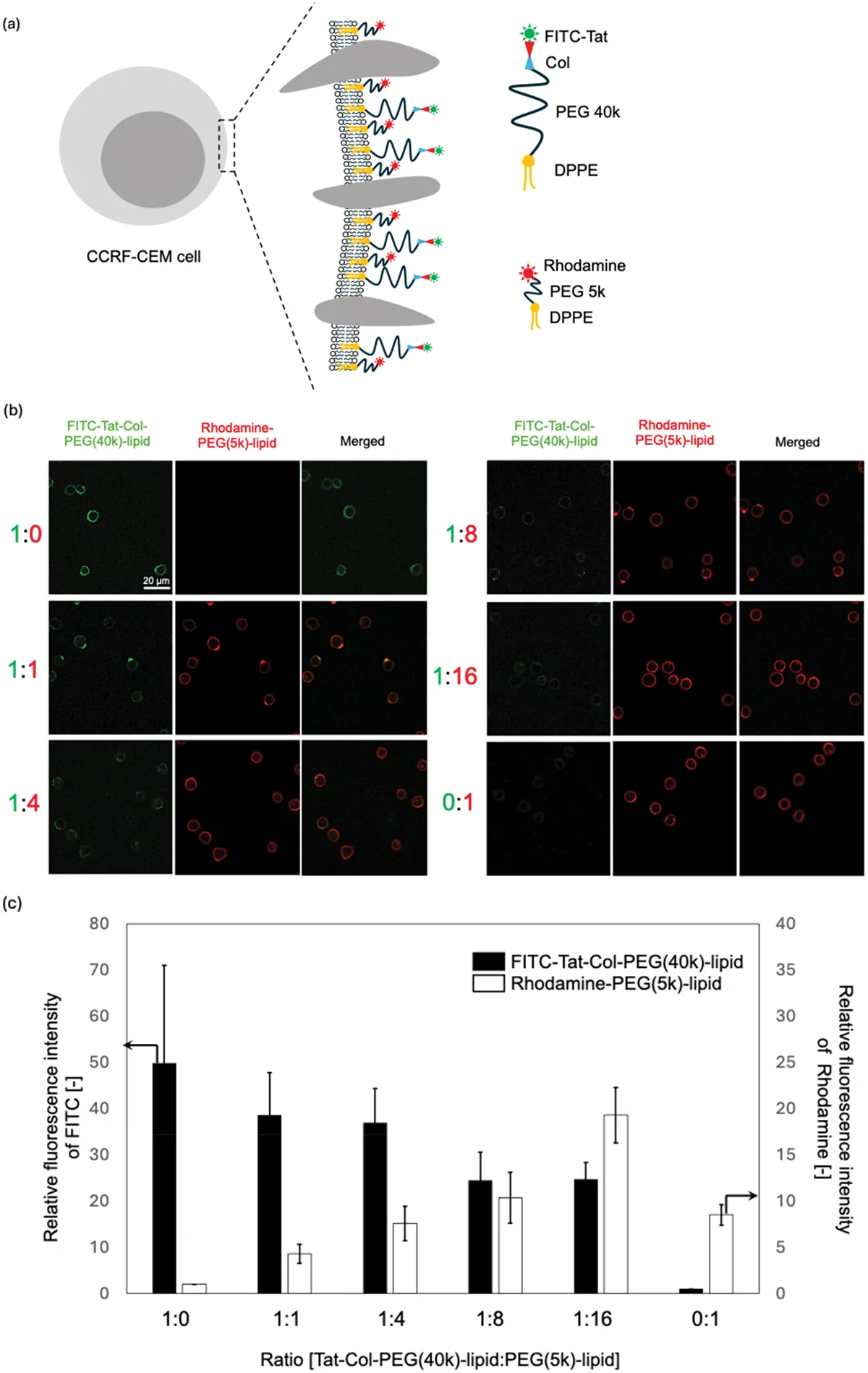
Co-insertion of Tat-Col-PEG(40k)-lipid and PEG(5k)-lipid into CCRF-CEM cell membranes. (a) Schematic illustration of membrane modification using FITC-labeled Tat-Col-PEG(40k)-lipid and Rhodamine-labeled PEG(5k)-lipid. (b) Confocal laser scanning microscopy images of CCRF-CEM cells after incubation with FITC-Tat-Col-PEG(40k)-lipid and Rhodamine-PEG(5k)-lipid at different input ratios. Green fluorescence indicates FITC-Tat-Col-PEG(40k)-lipid, red fluorescence indicates Rhodamine-PEG(5k)-lipid, and merged images show co-localization on the cell membrane. Scale bar: 20 µm. (c) Quantification of FITC and Rhodamine fluorescence intensities from modified CCRF-CEM cells by a fluorophotometer. FITC and Rhodamine fluorescence intensities in modified cells were normalized to those obtained under the 1:0 and 0:1 conditions, respectively, after correction for background signals from non-modified cells. Relative FITC and Rhodamine fluorescence intensities are shown as black bars on the left y-axis and white bars on the right y-axis, respectively. Data are presented as mean ± SD.    .

The incorporation of Tat-Col-PEG(40k)-lipids and PEG(5k)-lipids into the CCRF-CEM cell membranes was examined by confocal laser scanning microscopy. Here, FITC-labeled Tat-Col-PEG(40k)-lipid and Rhodamine-labeled PEG(5k)-lipid were used to visualize the distribution of each lipid component on the cell surface (Figure 1(a)). The molar ratios of the mixed solution of FITC-Tat-Col-PEG(40k)-lipids and Rhodamine-PEG(5k)-lipids were 1:0, 1:1, 1:4, 1:8, 1:16, and 0:1. After 30 min of incubation with the CCRF-CEM cells, fluorescence signals were mainly detected along the cell periphery under all conditions, indicating that the PEG-lipid conjugates were localized at the cellular membrane. In the 1:0 condition, a clear ring-shaped FITC fluorescence was observed, whereas a similar ring-shaped Rhodamine fluorescence was observed in the 0:1 condition, indicating that FITC-Tat-Col-PEG(40k)-lipid and Rhodamine-PEG(5k)-lipid were incorporated into the cellular membrane in a similar manner. Under mixed conditions, both FITC and rhodamine fluorescence signals were detected in the same cells, although their fluorescence intensities depended on the mixing ratio, confirming the co-insertion of Tat-Col-PEG(40k)-lipid and PEG(5k)-lipid. As the input ratio of Rhodamine-PEG(5k)-lipid increased, rhodamine fluorescence became more pronounced, whereas the FITC signal from the Tat-Col-PEG(40k)-lipid decreased accordingly (Figure 1(b)). This ratio-dependent change suggests that PEG(5k)-lipid competes with Tat-Col-PEG(40k)-lipid during membrane insertion, and that the surface composition of PEG(5k)-lipid and Tat-Col-PEG(40k)-lipid can be adjusted by premixing the two lipids.

### Quantitative analysis of modified cells

Ratio-dependent incorporation of the two PEG-lipid species was quantitatively evaluated using fluorescence intensity measurements and zeta potential analysis. Consistent with the confocal images, FITC fluorescence intensity was highest in cells treated with FITC-Tat-Col-PEG(40k)-lipids alone and decreased as the proportion of Rhodamine-PEG(5k)-lipids increased. In contrast, the rhodamine fluorescence intensity increased with increasing PEG(5k)-lipid content and became dominant under spacer-rich conditions (Figure 1(c)).

**Figure 2.**
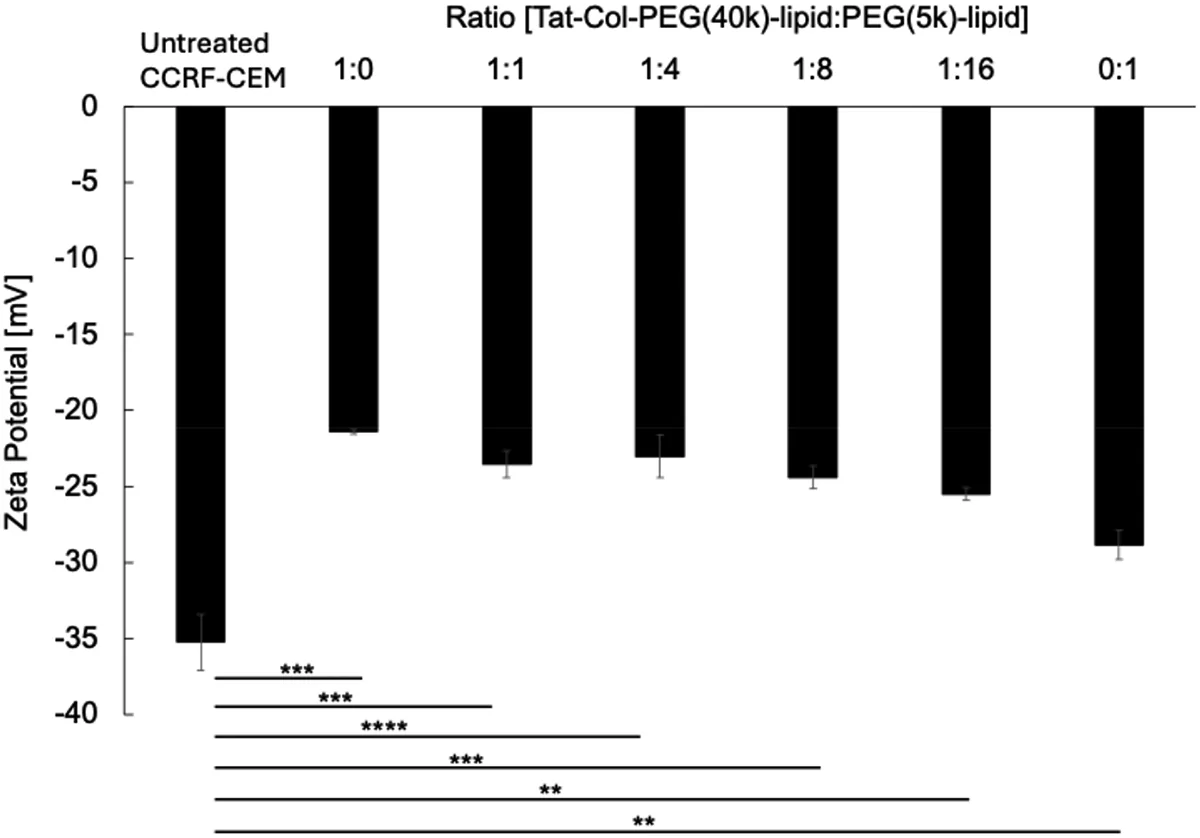
Zeta potential of CCRF-CEM cells after PEG-lipid modification. Cells were treated with Tat-Col-PEG(40k)-lipid and/or PEG(5k)-lipid at different input ratios. Untreated CCRF-CEM cells were used as a control. Data are presented as mean ± SD. n.S., not significant; *p < 0.05; **p < 0.01; ***p < 0.001; ****p < 0.0001.

Zeta potential measurements showed a corresponding change in the cell surface charge (Figure 2). Untreated CCRF-CEM cells exhibited a strongly negative surface potential, whereas Tat-Col-PEG(40k)-lipid modification shifted the zeta potential to a less negative value (~ −10 mV), consistent with the presence of positively charged Tat peptide on the cell surface. Increasing the PEG(5k)-lipid ratio gradually restored the more negative surface potential, suggesting the dilution and/or shielding of Tat peptides by the PEG layer. Together, these results indicate that the composition and surface properties of PEG-lipid-modified CCRF-CEM cells can be tuned by adjusting the input ratio of the two lipids.

The effect of buffer composition on collagenase activity was examined by comparing PBS and HBSS (Supplementary Information, Figure S1). HBSS produced more detached cells than PBS at the same collagenase concentration when the adhered cells were treated with collagenase in the buffer. Limited detachment was observed under control conditions without collagenase. These results indicate that HBSS provides a more suitable environment for collagenase-mediated cleavage of the Col linker, likely because of its physiological ionic composition and support of collagenase activity. Therefore, HBSS was used as the detachment buffer in subsequent experiments.

### Mixed ratio-dependent changes in adhesion morphology of modified CCRF-CEM cells

**Figure 3.**
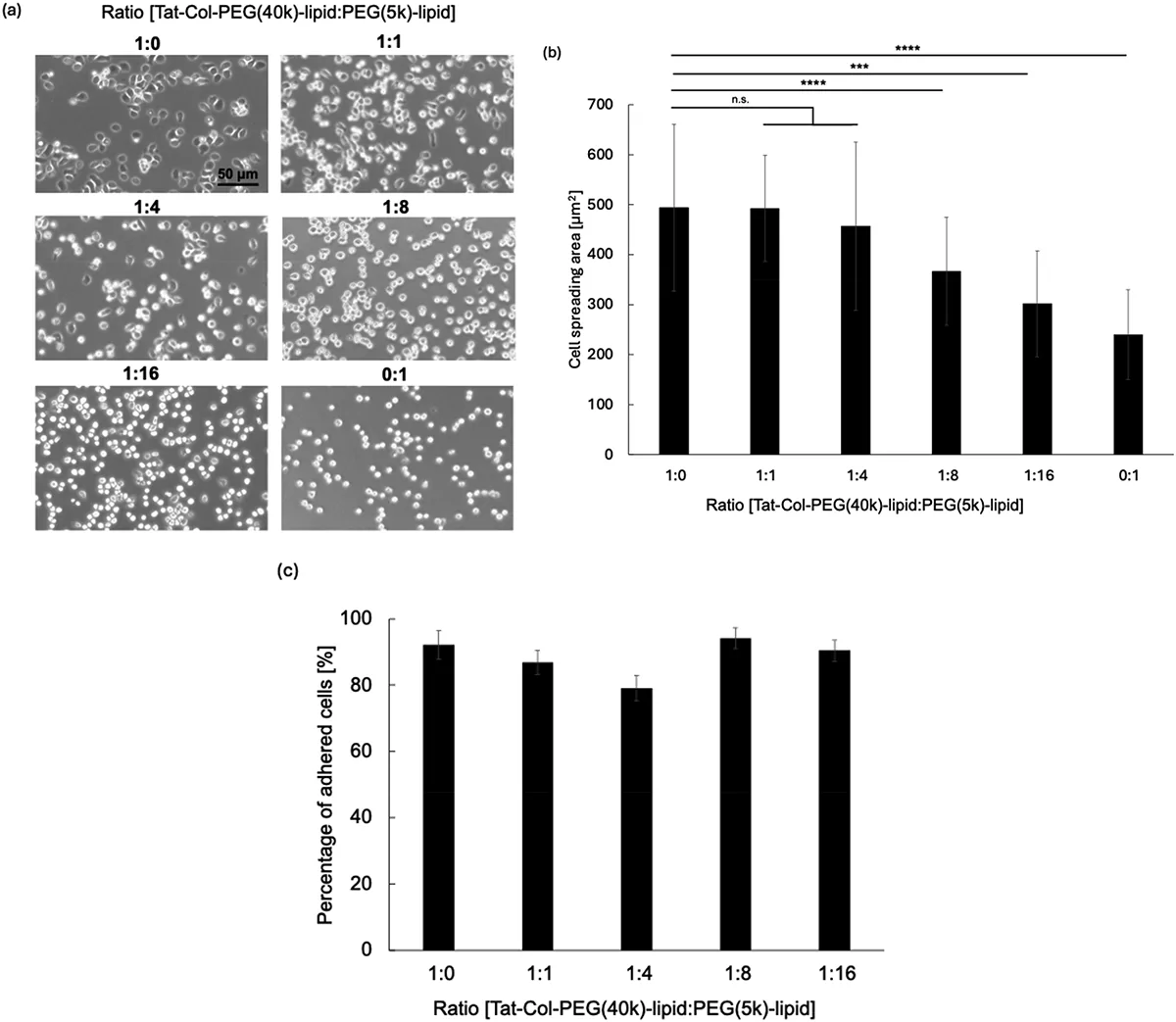
Ratio-dependent cell morphology change of PEG-lipid-modified CCRF-CEM cells on TCPS. (a) Phase-contrast microscopy images of CCRF-CEM cells modified with Tat-Col-PEG(40k)-lipid and PEG(5k)-lipid at different input ratios. Scale bar: 50 µm. (b) Quantification of cell spreading area after adhesion. (c) Adhesion efficiency of CCRF-CEM cells modified with Tat-Col-PEG(40k)-lipid and PEG(5k)-lipid at different input ratios. Data are presented as mean ± SD. n.S., not significant; *p < 0.05; **p < 0.01; ***p < 0.001; ****p < 0.0001.

The effect of the PEG-lipid composition on CCRF-CEM cell adhesion was evaluated on TCPS surfaces (Figure 3(a)). The cells were then washed with the buffer. CCRF-CEM cells modified with Tat-Col-PEG (40k)-lipids alone showed clear attachment to the TCPS surface and displayed a well-spread morphology, whereas cells treated with PEG(5k)-lipids alone remained mostly round and nonadherent, floating on the TCPS surface (Figure 3(a)).

When PEG(5k)-lipids were co-inserted at ratios of 1:1, 1:4, 1:8, and 1:16, treated cells adhered via Tat-Col-PEG(40k)-lipid-mediated interactions. As the input ratio of PEG(5k)-lipids increased, the morphology of the adhered cells gradually changed and the cells became more rounded. Although most cells displayed a round shape at ratios of 1:8–1:16, they firmly adhered to the TCPS surface.

From the imaging analysis of the cell area, the cells in the 1:0 and 1:1 groups showed the largest spreading areas, whereas increasing the PEG (5k)-lipid ratio generally reduced the cell spreading area (Figure 3(b)). This trend was particularly evident at higher PEG(5k)-lipid ratios, where the cells appeared less flattened and more rounded. The PEG(5k)-lipid group (0:1 ratio) showed the smallest apparent spreading area, which was consistent with a lack of adhesion. This result indicated that sufficient Tat-Col-PEG(40k)-lipids remained available to mediate cell–substrate interactions at these input ratios. The adhesion efficiency was approximately 80–90% for all Tat-Col-PEG(40k)-lipid-containing groups (Figure 3(c)), indicating that although the cell adhesion morphology was influenced by the co-insertion of PEG(5k)-lipids, the Tat-Col-PEG(40k)-lipid-mediated adhesion itself was not markedly affected. Therefore, it was possible to tune the adhesion behavior of CCRF-CEM cells by adjusting the mixing ratios of the two lipids.

### Effect of PEG(5k)-lipid ratio on collagenase-mediated detachment and cell viability

**Figure 4.**
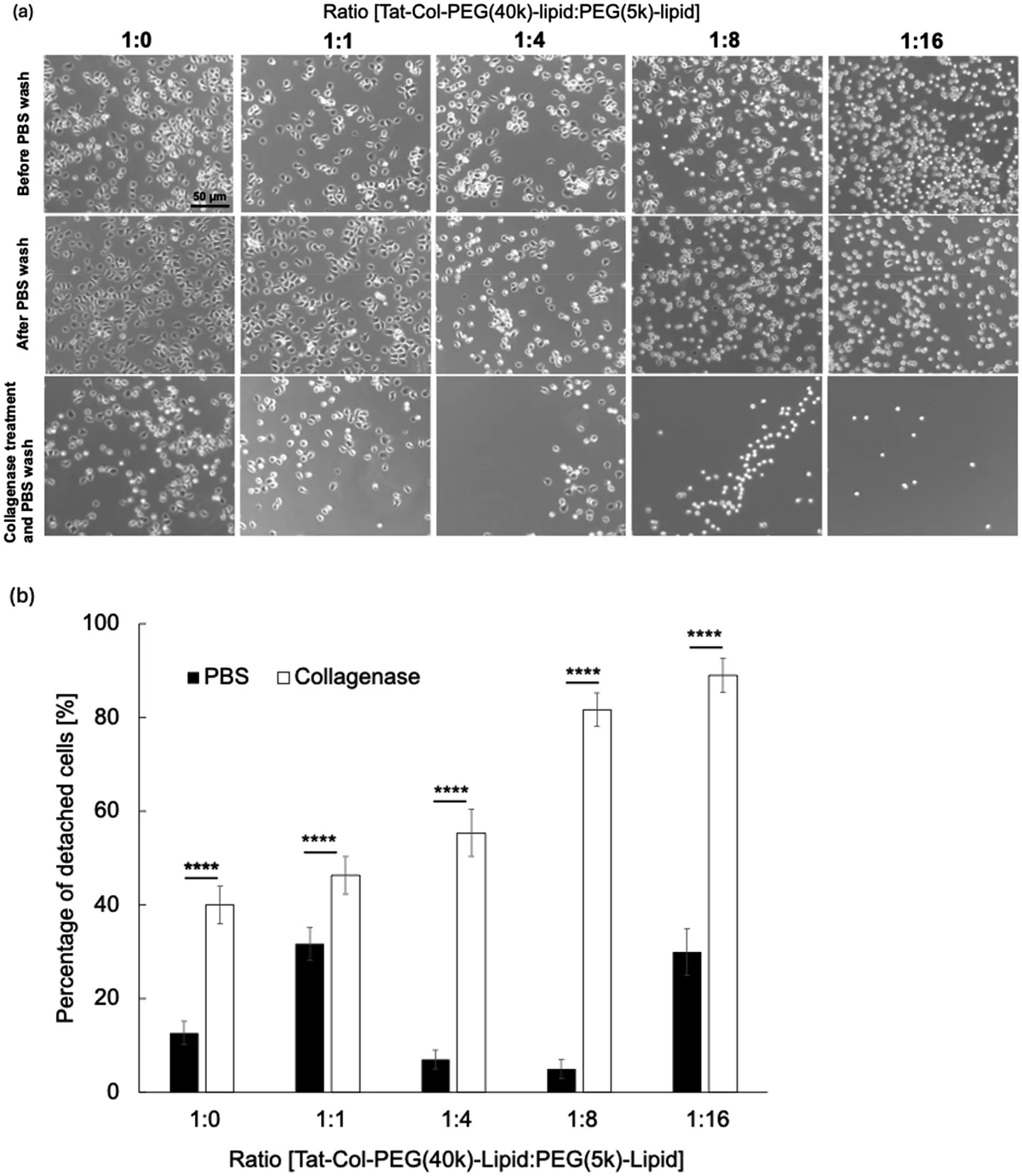
Collagenase-mediated detachment of PEG-lipid-modified CCRF-CEM cells. (a) Phase-contrast microscopy images of PEG-lipid-modified CCRF-CEM cells before washing, after PBS washing, and after collagenase treatment followed by washing. Cells were modified with Tat-Col-PEG(40k)-lipid and PEG(5k)-lipid at ratios of 1:0, 1:1, 1:4, 1:8, and 1:16. PBS washing served as a non-enzymatic washing to compare with collagenase treatment. Scale bar: 50 µm. (b) Quantification of detached cells after PBS or collagenase treatment. Data are presented as mean ± SD. n.S., not significant; *p < 0.05; **p < 0.01; ***p < 0.001; ****p < 0.0001.

Next, collagenase-mediated detachment of the adhered cells was evaluated. Cells modified with Tat-Col-PEG(40k)-lipids and PEG(5k)-lipids in different ratios were first allowed to adhere to the TCPS surface, followed by washing with PBS as a control or collagenase in HBSS (Figure 4(a)).

The detachment ratio was calculated for each input mixture of the two lipids from the images obtained at each step. Quantification confirmed that collagenase treatment significantly increased cell detachment compared to PBS washing at all tested ratios (Figure 4(b)).

In the absence of PEG(5k)-lipid at a 1:0 ratio, approximately 40% of the adhered cells were harvested by collagenase treatment, indicating that collagenase could not access the Col domain. Presumably, Tat-Col-PEG(40k)-lipids were highly dense on the cell surface and collagenase access was blocked by steric hindrance. However, increasing the PEG(5k)-lipid ratio enhanced collagenase-mediated cell detachment, with the most efficient detachment observed in the 1:8 and 1:16 groups. These results suggest that PEG(5k)-lipid acts as a spacer that improves the access of collagenase to the cleavable Col domain while maintaining sufficient Tat-mediated cell adhesion before collagenase treatment.

**Figure 5.**
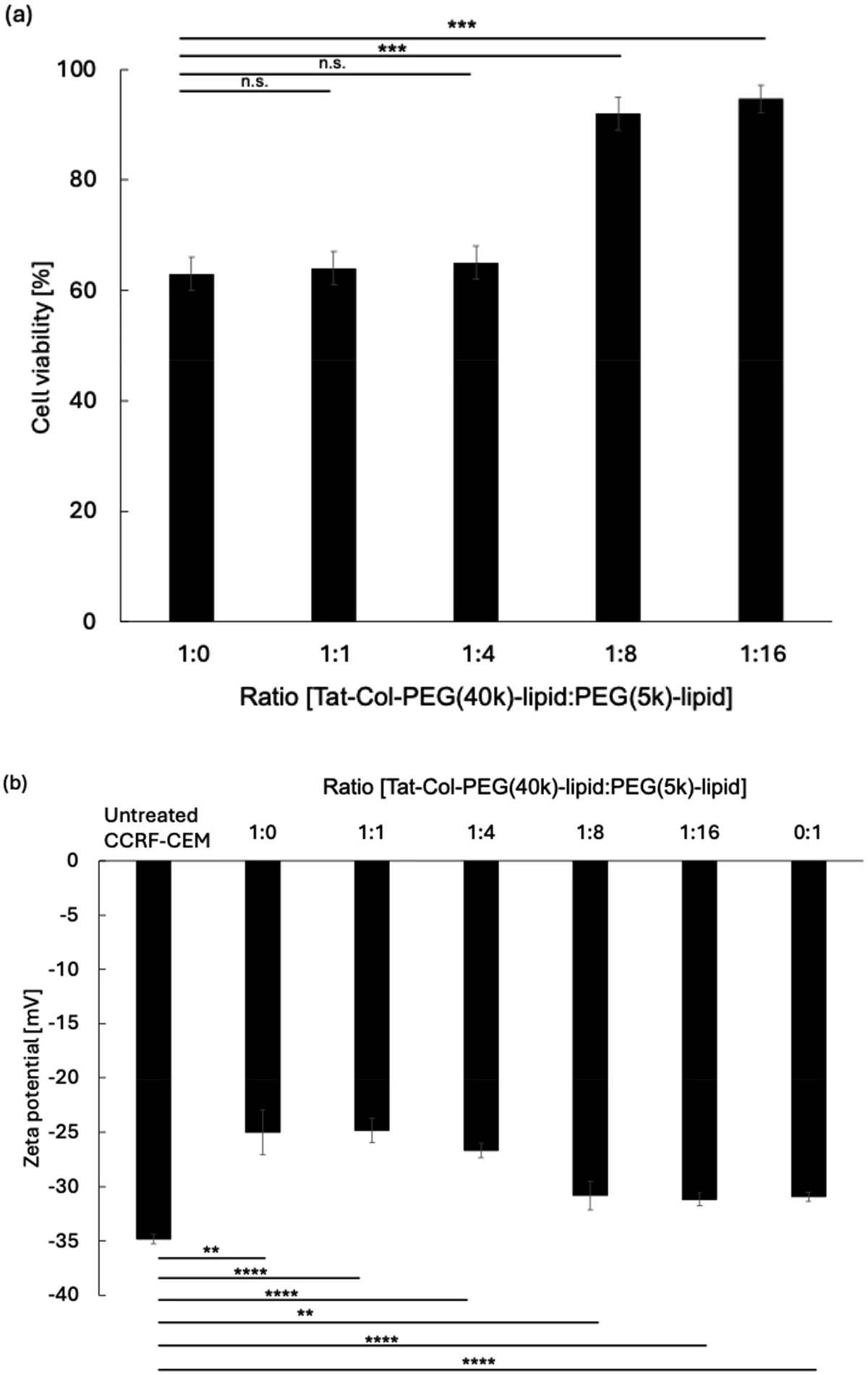
Viability and zeta potential of CCRF-CEM cells after collagenase-mediated detachment. (a) Viability of detached CCRF-CEM cells from TCPS surfaces after collagenase treatment. (b) Zeta potential of CCRF-CEM cells after collagenase treatment and 12 h culture. Untreated CCRF-CEM cells and PEG(5k)-lipid-modified cells were included as controls. Data are presented as mean ± SD. n.S., not significant; *p < 0.05; **p < 0.01; ***p < 0.001; ****p < 0.0001.

The viability of the detached cells was then examined. The viability of cells detached from the 1:0, 1:1, and 1:4 groups was approximately 60% for three groups, whereas it markedly increased in the 1:8 and 1:16 groups, reaching above 90% (Figure 5(a)). We considered that this improvement reflected the reduced membrane stress caused by the co-insertion of PEG(5k)-lipids with Tat-Col-PEG(40k)-lipids at higher ratios. Cells may undergo stress during enzymatic detachment, particularly when cell adhesion is predominantly mediated by the Tat peptide at low PEG(5k)-lipid ratios. Therefore, the co-insertion of PEG(5k)-lipids can enhance the detachment efficiency while maintaining high cell viability.

To further assess the post-detachment cell-surface state, the zeta potential was measured after collagenase treatment (Figure 5(b)). The zeta potential of the detached cells remained less negative than that of the untreated CCRF-CEM cells in the 1:0, 1:1, and 1:4 groups, whereas the values were closer to those of the PEG(5k)-lipid-only condition in the 1:8 and 1:16 groups. This suggests that spacer-rich formulations reduce the residual Tat-associated surface charge after detachment. Together, these results indicate that increasing the PEG(5k)-lipid ratio improved collagenase-mediated release and post-detachment viability, with 1:8 and 1:16 providing the most favorable detachment performance among the tested conditions.

### Transient adhesion of CCRF-CEM cells on a fibrous scaffold by PEG-lipids

**Figure 6.**
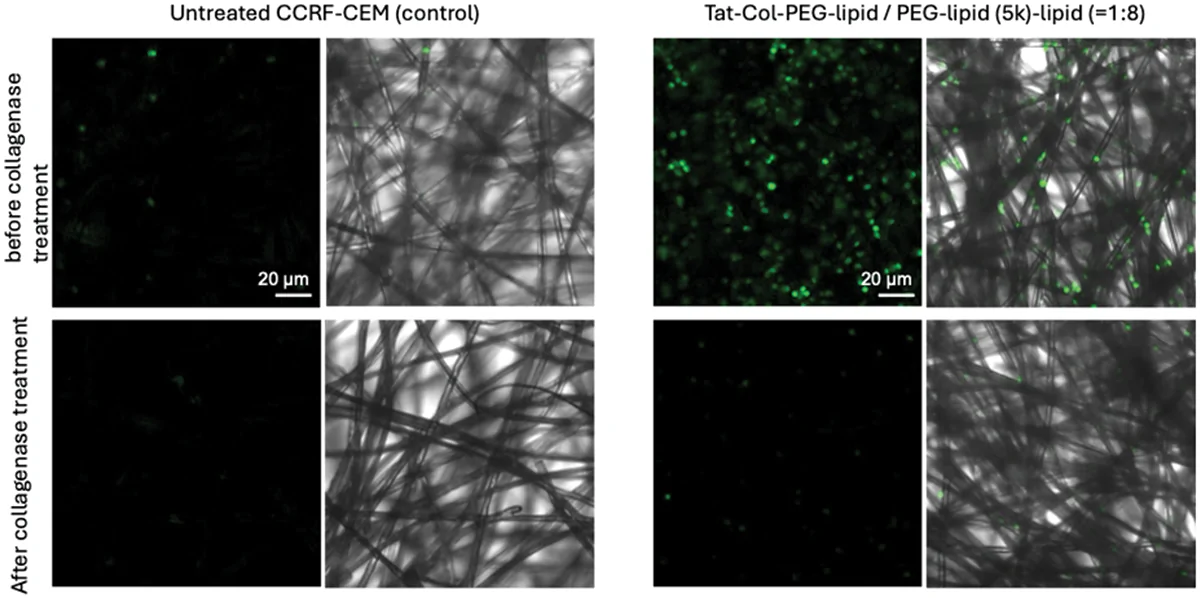
Adhesion and collagenase-mediated detachment of PEG-lipid-modified CCRF-CEM cells on nonwoven fibrous scaffolds. Fluorescence and merged fluorescence/bright-field images of CCRF-CEM cells on a nonwoven fibrous scaffold after cell treatment and after collagenase-mediated detachment. Green fluorescence indicates cells labeled with CellTrace™ Oregon Green. Scale bars: 20 µm.

Finally, the mixed PEG-lipids were applied to a non-woven fibrous scaffold to examine whether the transient adhesion strategy could be extended to a three-dimensional substrate (Figure 6). The control cells showed only limited attachment to the scaffold, and almost no cells were observed. In contrast, when the cells were modified with FITC-Tat-Col-PEG(40k)-lipids and PEG(5k)-lipids (1:8 ratio), fluorescently labeled cells were observed on the fibrous structure, indicating efficient cell attachment to the scaffold via Tat-mediated cell adhesion. Merged fluorescence and bright-field images confirmed that the modified cells were distributed along the fibers. After collagenase treatment, the number of cells adhered to the scaffold was markedly reduced, suggesting that most cells were released from the fibrous substrate. These results indicate that collagenase-responsive detachment can be achieved not only on a 2D substrate surface but also within a structurally complex three-dimensional scaffold.

Thus, the mixed PEG-lipid modification strategy can be applied for transient cell immobilization and recovery in scaffold-based culture systems or cell-processing platforms.

## Discussion

This study presents a cell-surface engineering strategy that allows suspended CCRF-CEM cells to attach to substrates and be released on demand by collagenase treatment. Suspension immune and leukemia-derived cells are widely used in biomedical research, gene delivery studies, and cell-based therapy development; however, their non-adherent nature makes their handling difficult [1,3–6]. We aimed to manipulate suspension cells by cell surface engineering, where synthetic polymers, nanoparticles, and lipid anchors were used to add temporary functions to living cells without genetic modification [8,10–15,19,20]. By enabling transient substrate immobilization and enzyme-triggered recovery, this strategy may provide a practical platform for improving nucleic acid delivery and downstream handling of suspension cells. Such a workflow could bridge the operational gap between suspension and adherent cell cultures and may be incorporated into routine laboratory procedures.

Our system separates three functions within one surface coating: membrane anchoring by the lipid, spacing by PEG, and the adhesive domain provided by the Tat peptide. PEG-lipid conjugates are spontaneously inserted into cell membranes through hydrophobic interactions, and related approaches have been used to display polymers, DNA, and other functional molecules on living cell surfaces [14–18]. Tat-Col-PEG-lipid provided adhesive activity, whereas PEG(5k)-lipid served as a spacer to adjust the density of the collagen-cleavable sites. This design is important because cell adhesion must be strong enough to survive washing but weak or accessible enough for detachment without damaging the cells.

Tat was chosen because its basic arginine-rich sequence can penetrate membranes despite being positively charged [23,24]. Previous studies have shown that CPP-PEG-lipids, including Tat-PEG-lipids, can induce spontaneous adhesion of suspension cells without substrate pretreatment [21,22]. In our previous report, we showed that Tat peptides positioned at the distal end of PEG-lipids were exposed on the cell surface, as biotinylated Tat peptides were clearly detected by fluorescently labeled streptavidin, which is not cell permeable [21]. Although a fraction of Tat peptides may have been internalized because of their cell-penetrating properties, the streptavidin-labeling result supports that a detectable population of Tat peptides remained accessible on the cell surface. This surface-exposed Tat population is likely responsible for mediating cell–substrate interactions in the present system.

We also previously reported that liposomes modified with Tat-Col-PEG-lipids spontaneously adhered to substrate surfaces and were subsequently detached by collagenase treatment, providing proof of concept for enzyme-responsive adhesion control [25]. In that liposome model, efficient detachment could be achieved using Tat-Col-PEG-lipids alone. However, the same formulation was insufficient for efficient detachment of living cells. This difference may arise from the much larger size and more complex surface architecture of living cells compared with liposomes. Whereas liposomes are small vesicles with relatively simple lipid bilayers, living cells possess heterogeneous membrane proteins, glycocalyx structures, and a deformable cell body, all of which may alter the cell–substrate interface and restrict collagenase access to the Col domain. Therefore, co-insertion of PEG(5k)-lipids was necessary to improve enzymatic accessibility and enable efficient release of living cells.

Adhesion of conventional adherent cells is primarily mediated by interactions between integrins and extracellular matrix proteins, such as fibronectin adsorbed from serum-containing medium. In contrast, suspension cells do not form stable integrin-mediated contacts with ordinary culture substrates and therefore remain suspended in culture medium. In the present system, treatment with Tat-PEG-lipids exposes the cationic Tat peptides on the cell surface, which likely promotes electrostatic interactions with negatively charged substrate surfaces or presumably adsorbed serum protein layers, thereby enabling cell immobilization. Although serum proteins from FBS are expected to adsorb onto the substrate surface, the Tat peptide can still interact with exposed regions of the substrate because of smaller molecular weight or protein layer, contributing to substrate attachment.

We previously reported that the half-life of Tat-PEG-lipid on the cell surface was for approximately 9 h [26]. Tat-PEG-lipid-modified cells gradually detached from the substrate during culture, with approximately half of the initially adhered cells detaching after one day [21]. These findings also indicate that Tat-PEG-lipid-induced adhesion is transient over time, even without enzymatic treatment. Our results are consistent with previous findings, while further introducing an enzyme-responsive release step through the collagenase-cleavable Col domain. Therefore, this system is not simply a ‘cell glue’; it acts as a tunable temporary glue. At higher PEG(5k)-lipid ratios, the cells showed reduced spreading, suggesting that spacer lipids weakened Tat-mediated cell–substrate contact without completely preventing adhesion. This weaker but stable contact appears to be favorable for later cell release. The detachment results showed that the co-insertion of PEG(5k)-lipids was important for balancing adhesion and detachment. A dense Tat-Col-PEG(40k)-lipid presentation produced strong adhesion; however, collagenase-mediated detachment was limited. This may be due to restricted enzyme access at the crowded cell–substrate interface. When Tat-Col-PEG(40k)-lipid was diluted with PEG(5k)-lipid, the Col domain likely became more accessible to collagenase, improving cleavage and subsequent cell release. The role of PEG(5k)-lipid can be interpreted as creating intermolecular spacing between Tat-Col-PEG(40k)-lipids on the cell surface, partly through the excluded-volume effect of the PEG chains. At the same time, the shorter PEG(5k) chain may be advantageous because it provides spacing without forming a steric barrier that would hinder collagenase access to the Col domain. In addition, our previous study suggested that Tat-Col-PEG(40k)-lipids can laterally diffuse within the cell membrane and accumulate at the cell–substrate interface, increasing the local density of Tat-Col-PEG(40k)-lipids at the adhesion site [22]. Such interfacial enrichment may further restrict collagenase access, explaining why densely modified cells showed limited enzymatic detachment. This interpretation is consistent with studies showing that the PEG spacer length and molecular mobility can affect cell capture and release efficiency [27,28]. This also agrees with the enzyme-cleavable peptide systems on polymeric microfibers, in which access to the cleavable sequence is critical for cell release.

The supplementary PEG chain length results further explain why Tat-Col-PEG(40k)-lipids were selected. Both Tat-Col-PEG(20k)-lipid and Tat-Col-PEG(40k)-lipid supported high initial adhesion with or without PEG(5k)-lipid (Supplementary Information, Figure S2a). However, their release behaviors differed. In the case of Tat-Col-PEG(20k)-lipid, adding PEG(5k)-lipid increased collagenase-mediated detachment but also increased PBS-treated release, suggesting weaker adhesion and higher sensitivity to non-enzymatic washing. In contrast, Tat-Col-PEG(40k)-lipid treatment maintained lower cell detachment in PBS, while showing stronger collagenase-triggered release without affecting cell viability when PEG(5k)-lipid was co-inserted (Supplementary Information, Figure S2b, c). This agrees with earlier Tat-PEG-lipid studies showing that PEG chain length affects Tat presentation, cell spreading, and adhesion strength [21,22].

The zeta potential results after collagenase treatment were also noteworthy. Although collagenase treatment and subsequent culturing shifted the surface charge toward the untreated state, the zeta potential did not fully return to that of native CCRF-CEM cells. This does not contradict the transient nature of this modification. This suggests that the surface recovery requires a long time. Some PEG-lipid fragments remained on the membrane and gradually disappeared after detachment from the cell surface. Physically inserted lipid-based modifications have shorter retention times than covalent modifications; however, they are not removed immediately [13,20]. Therefore, the modification should be described as transient but not instantly erased. After detachment, a cell culture may be required to determine when the surface potential fully returns to its native range.

When cells were modified with Tat-Col-PEG(40k)-lipid, the zeta potential shifted to a less negative value (~−10 mV) but remained negative, reflecting the net surface charge of the entire cell. This result suggests that although positively charged Tat peptides were locally displayed on the cell surface, their contribution was averaged together with negatively charged membrane components, including membrane proteins and glycocalyx structures. Therefore, a negative average zeta potential does not necessarily exclude local Tat-mediated interactions at the cell–substrate interface. When modified cells contact a substrate surface, exposed Tat peptides can locally mediate electrostatic interactions with negatively charged substrate regions or adsorbed protein layers, leading to cell attachment. Conversely, if modified cells encounter each other before settling onto the substrate, the same local Tat-mediated interactions may promote cell–cell aggregation, as previously reported for CPP-PEG-lipid-modified cells [26].

Finally, this strategy is not limited to cell culture in 2D culture dishes. PEG-lipid-modified CCRF-CEM cells attached to a nonwoven fibrous 3D scaffold were harvested after collagenase treatment, indicating that the same transient adhesion principle can be applied to a more complex three-dimensional substrate. This could be useful for the temporary immobilization of suspended cells in scaffold-based culture, cell processing, or gene delivery workflows. Similar capture-and-release concepts have also been used in microfiber- and light-responsive hydrogel systems, supporting the broader value of recoverable cell-immobilization platforms [28,29]. In the photodegradable hydrogel system, antibodies against specific cell-surface receptors were immobilized within photocleavable hydrogels to capture target cells, and the captured cells were subsequently released by light irradiation. This approach is useful for selective cell collection using monoclonal antibodies; however, it requires the preparation of photocleavable hydrogels and antibody immobilization, which may limit its routine applicability. In contrast, the present PEG-lipid-based strategy can be applied to standard culture dishes and glass surfaces without major changes to the experimental setup. Cell adhesion and recovery can be achieved simply by treating cells with Tat-Col-PEG(40k)-lipids and subsequently applying collagenase for cell harvesting. Thus, compared with substrate-engineering approaches, our method shifts the functionalization step from the material surface to the cell surface, enabling transient adhesion and on-demand recovery using conventional culture substrates.

## Conclusion

Co-assembly of functional Tat-Col-PEG(40k)-lipids with non-functional PEG(5k)-lipids allowed precise tuning of the surface density, resulting in a balance between stable adhesion and efficient enzymatic detachment. Notably, PEG(5k)-lipids played a critical role, with higher ratios providing higher adhesion efficiency, enzyme-triggered release, and cell viability than the lower ratios. The optimized mixed PEG-lipids exhibited a robust performance across different substrates, including planar surfaces and fibrous scaffolds.

## Supplementary Material

Supplemental Material

## References

[1] Drexler HG, MacLeod RA. Leukemia-lymphoma cell lines as model systems for hematopoietic research. Ann Med. 2003;35(6):404–15. doi: 10.1080/07853890310012094 14572164

[2] Foley GE, Lazarus H, Farber S, et al. Continuous culture of human Lymphoblasts from peripheral blood of a child with acute leukemia. Cancer. 1965;18:522–529. doi: 10.1002/1097-0142(196504)18:4<522:aid-cncr2820180418>3.0.co;2-j 14278051

[3] Larsen HO, Roug AS, Nielsen K, et al. Nonviral transfection of leukemic primary cells and cells lines by siRNA-a direct comparison between Nucleofection and accell delivery. Exp Hematol. 2011;39(11):1081–1089. doi: 10.1016/j.exphem.2011.08.003 21856272

[4] Kardani K, Milani A, Bolhassani A. Gene delivery in adherent and suspension cells using the combined physical methods. Cytotechnology. 2022;74(2):245–257. doi: 10.1007/s10616-022-00524-4 35464169 PMC8975990

[5] Reagan JL, Fast LD, Safran H, et al. Cellular immunotherapy for refractory hematological malignancies. J Transl Med. 2013;11:150. doi: 10.1186/1479-5876-11-150 23782682 PMC3689050

[6] Wang S, Tian D. High transfection efficiency and cell viability of immune cells with nanomaterials-based transfection reagent. Biotechniques. 2022;72(5):219–224. doi: 10.2144/btn-2022-0024 35369729

[7] Wang Y, Yang Y, Wang X, et al. The varied influences of cell adhesion and spreading on gene transfection of mesenchymal stem cells on a micropatterned substrate. Acta Biomater. 2021;125:100–111. doi: 10.1016/j.actbio.2021.01.042 33524558

[8] Stephan MT, Irvine DJ. Enhancing cell therapies from the outside in: cell surface engineering using synthetic nanomaterials. Nano Today. 2011;6(3):309–325. doi: 10.1016/j.nantod.2011.04.001 21826117 PMC3148657

[9] Ayer M, Burri O, Guiet R, et al. Biotin-NeutrAvidin mediated immobilization of polymer micro- and nanoparticles on T lymphocytes. Bioconjug Chem. 2021;32(3):541–552. doi: 10.1021/acs.bioconjchem.1c00026 33621057

[10] Park J, Andrade B, Seo Y, et al. Engineering the surface of therapeutic “living” cells. Chem Rev. 2018;118(4):1664–1690. doi: 10.1021/acs.chemrev.7b00157 29336552 PMC8243527

[11] Arno MC. Engineering the mammalian cell surface with synthetic polymers: strategies and applications. Macromol Rapid Commun. 2020;41(18):e2000302. doi: 10.1002/marc.202000302 32803807

[12] Yang H, Yao L, Wang Y, et al. Advancing cell surface modification in mammalian cells with synthetic molecules. Chem Sci. 2023;14(46):13325–13345. doi: 10.1039/d3sc04597h 38033886 PMC10685406

[13] Kim S, Kim K. Lipid-mediated ex vivo cell surface engineering for augmented cellular functionalities. Biomater Adv. 2022;140:213059. doi: 10.1016/j.bioadv.2022.213059 35961186

[14] Teramura Y, Iwata H. Cell surface modification with polymers for biomedical studies. Soft Matter. 2010;6(6):1081–1091. doi: 10.1039/b913621e

[15] Teramura Y, Ekdahl KN, Fromell K, et al. Potential of cell surface engineering with biocompatible polymers for biomedical applications. Langmuir. 2020;36(41):12088–12106. doi: 10.1021/acs.langmuir.0c01678 32927948

[16] Teramura Y, Kaneda Y, Iwata H. Islet-encapsulation in ultra-thin layer-by-layer membranes of poly(vinyl alcohol) anchored to poly(ethylene glycol)-lipids in the cell membrane. Biomaterials. 2007;28(32):4818–4825. doi: 10.1016/j.biomaterials.2007.07.050 17698188

[17] Teramura Y, Chen H, Kawamoto T, et al. Control of cell attachment through polyDNA hybridization. Biomaterials. 2010;31(8):2229–2235. doi: 10.1016/j.biomaterials.2009.11.098 20004971

[18] Teramura Y. Cell surface modification with ssDNA-PEG-lipid for analysing intercellular interactions between different cells. Biomaterials. 2015;48:119–128. doi: 10.1016/j.biomaterials.2015.01.032 25701037

[19] Wang X, Wen C, Davis B, et al. Synthetic DNA for cell surface engineering: experimental comparison between click conjugation and lipid insertion in terms of cell viability, engineering efficiency, and displaying stability. ACS Appl Mater Interface. 2022;14(3):3900–3909. doi: 10.1021/acsami.1c22774 PMC12056530 35020367

[20] Rabuka D, Forstner MB, Groves JT, et al. Noncovalent cell surface engineering: incorporation of bioactive synthetic glycopolymers into cellular membranes. J Am Chem Soc. 2008;130(18):5947–5953. doi: 10.1021/ja710644g 18402449 PMC2724873

[21] Teramura Y, Asif S, Ekdahl KN, et al. Cell adhesion induced using surface modification with cell-penetrating peptide-conjugated poly(ethylene glycol)-lipid: a new cell glue for 3D cell-based structures. ACS Appl Mater Interface. 2017;9(1):244–254. doi: 10.1021/acsami.6b14584 27976850

[22] Noiri M, Goto Y, Sato Y, et al. Exogenous cell surface modification with cell penetrating peptide-conjugated lipids causes spontaneous cell adhesion. ACS Appl Bio Mater. 2021;4(5):4598–4606. doi: 10.1021/acsabm.1c00335 35006797

[23] Vives E, Brodin P, Lebleu B. A truncated HIV-1 Tat protein basic domain rapidly translocates through the plasma membrane and accumulates in the cell nucleus. J Biol Chem. 1997;272(25):16010–16017. doi: 10.1074/jbc.272.25.16010 9188504

[24] Wender PA, Mitchell DJ, Pattabiraman K, et al. The design, synthesis, and evaluation of molecules that enable or enhance cellular uptake: peptoid molecular transporters. Proc Natl Acad Sci U S A. 2000;97(24):13003–13008. doi: 10.1073/pnas.97.24.13003 11087855 PMC27168

[25] Sato Y, Asawa K, Huang T, et al. Induction of spontaneous liposome adsorption by exogenous surface modification with cell-penetrating peptide-conjugated lipids. Langmuir. 2021;37(32):9711–9723. doi: 10.1021/acs.langmuir.1c01072 34342462

[26] Sato Y, Chung UI, Teramura Y. Induction of intercellular interaction and cell fusion by cell-penetrating peptide-conjugated lipids. J Mater Chem B. 2025;13(39):12546–12556. doi: 10.1039/d5tb01318f 40891702

[27] Yoshihara A, Sekine R, Yamada Y, et al. Study on polyethylene glycol cross-linker in peptide-conjugated antibody on efficiency of cell capture and release. Anal Biochem. 2020;602:113790. doi: 10.1016/j.ab.2020.113790 32470345

[28] Yoshihara A, Sekine R, Ueki T, et al. Rapid and highly efficient capture and release of cancer cells using polymeric microfibers immobilized with enzyme-cleavable peptides. Acta Biomater. 2018;67:32–41. doi: 10.1016/j.actbio.2017.11.055 29223702

[29] Shin DS, You J, Rahimian A, et al. Photodegradable hydrogels for capture, detection, and release of live cells. Angew Chem Int Ed Engl. 2014;53(31):8221–8224. doi: 10.1002/anie.201404323 24931301 PMC4380505

