# Supplementary material for: Spatial control of PEG-lipid interfaces at cell surface enables on-demand adhesion and enzymatic detachment of suspension cells
Source: Sci Technol Adv Mater. 2026 Aug 7;27(1):2713991. doi: 10.1080/14686996.2026.2713991 (PMC13508557; doi:10.1080/14686996.2026.2713991)
Supplement: Supplementary file 1 [file TSTA_A_2713991_SM7372.docx]

**Supporting Information**

**Spatial control of PEG-lipid interfaces at cell surface enables on-demand adhesion and enzymatic detachment of suspension cells**

Zhihan Yang1, Yuya Sato2, Yuji Teramura1,2*, Tadashi Nakaji-Hirabayashi3,4,5*

^1^Master’s/Doctoral Program in Life Science Innovation (T-LSI), University of Tsukuba, 1-1-1 Tennodai, Tsukuba, Ibaraki, 305-8577, Japan

^2^Cellular and Molecular Biotechnology Research Institute (CMB), National Institute of Advanced Industrial Science and Technology (AIST), AIST Tsukuba Central 5, 1-1-1 Higashi, Tsukuba, Ibaraki 305-8565, Japan

^3^Faculty of Engineering, Academic Assembly, University of Toyama, Toyama, Japan

^4^Graduate School of Science and Engineering, University of Toyama, Toyama, Japan

^5^Interdisciplinary Graduate School of Medicine, Pharmacy, Science and Engineering, University of Toyama, Toyama, Japan

**CONTACT**

Yuji Teramura

Tel: +81(0)80-2184-0654, E-mail: [y.teramura@aist.go.jp](mailto:y.teramura@aist.go.jp)

Tadashi Nakaji-Hirabayashi

Tel: +81(0)76-445-6011, E-mail: nakaji@eng.u-toyama.ac.jp

**Figure S1.** Collagenase-mediated detachment of PEG-lipid-modified CCRF-CEM cells with different concentrations (50 and 100 U/mL) and solutions (PBS and HBSS). Data are presented as mean ± SD. n.s., not significant; *****p* < 0.0001.

**Figure S2.** (a) Adhesion efficiency of CCRF-CEM cells modified with Tat-Col-PEG(20k, 40k)-lipid combining with and without PEG(5k)-lipid. (b) Collagenase-mediated detachment of PEG-lipid-modified CCRF-CEM cells with different PEG length (20k, 40k). (c) Viability of detached CCRF-CEM cells from TCPS surfaces after collagenase treatment. Data are presented as mean ± SD. n.s., not significant; ***p* < 0.01; ****p* < 0.001.
